# Radiology AI and sustainability paradox: environmental, economic, and social dimensions

**DOI:** 10.1186/s13244-025-01962-2

**Published:** 2025-04-17

**Authors:** Burak Kocak, Andrea Ponsiglione, Valeria Romeo, Lorenzo Ugga, Merel Huisman, Renato Cuocolo

**Affiliations:** 1 Department of Radiology, University of Health Sciences, Basaksehir Cam and Sakura City Hospital, Istanbul, Turkey; 2 Department of Advanced Biomedical Sciences, University of Naples Federico II, Naples, Italy; 3 Department of Advanced Medical and Surgical Sciences, University of Campania “Luigi Vanvitelli”, Naples, Italy; 4 Department of Radiology and Nuclear Medicine, Radboud University Medical Center, Nijmegen, The Netherlands; 5 Department of Medicine, Surgery and Dentistry, University of Salerno, Baronissi, Italy

**Keywords:** Artificial intelligence, Radiology, Sustainability, Environmental health, Health equity

## Abstract

**Abstract:**

Artificial intelligence (AI) is transforming radiology by improving diagnostic accuracy, streamlining workflows, and enhancing operational efficiency. However, these advancements come with significant sustainability challenges across environmental, economic, and social dimensions. AI systems, particularly deep learning models, require substantial computational resources, leading to high energy consumption, increased carbon emissions, and hardware waste. Data storage and cloud computing further exacerbate the environmental impact. Economically, the high costs of implementing AI tools often outweigh the demonstrated clinical benefits, raising concerns about their long-term viability and equity in healthcare systems. Socially, AI risks perpetuating healthcare disparities through biases in algorithms and unequal access to technology. On the other hand, AI has the potential to improve sustainability in healthcare by reducing low-value imaging, optimizing resource allocation, and improving energy efficiency in radiology departments. This review addresses the sustainability paradox of AI from a radiological perspective, exploring its environmental footprint, economic feasibility, and social implications. Strategies to mitigate these challenges are also discussed, alongside a call for action and directions for future research.

**Critical relevance statement:**

By adopting an informed and holistic approach, the radiology community can ensure that AI’s benefits are realized responsibly, balancing innovation with sustainability. This effort is essential to align technological advancements with environmental preservation, economic sustainability, and social equity.

**Key Points:**

- AI has an ambivalent potential, capable of both exacerbating global sustainability issues and offering increased productivity and accessibility.
- Addressing AI sustainability requires a broad perspective accounting for environmental impact, economic feasibility, and social implications.
- By embracing the duality of AI, the radiology community can adopt informed strategies at individual, institutional, and collective levels to maximize its benefits while minimizing negative impacts.

**Graphical Abstract:**

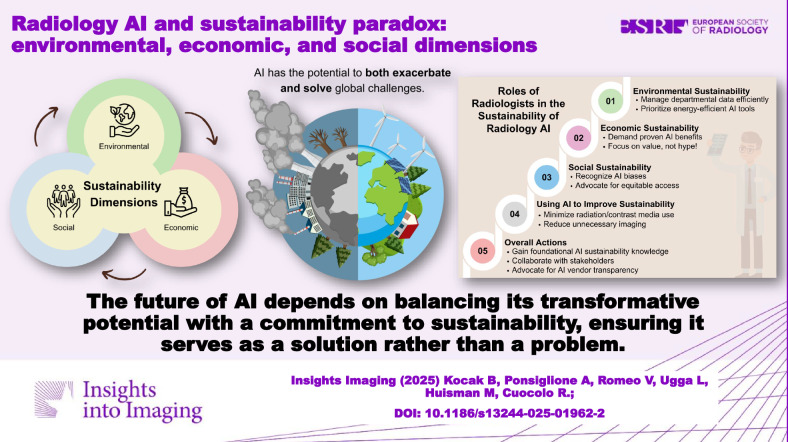

## Introduction

**Fig. 1:**
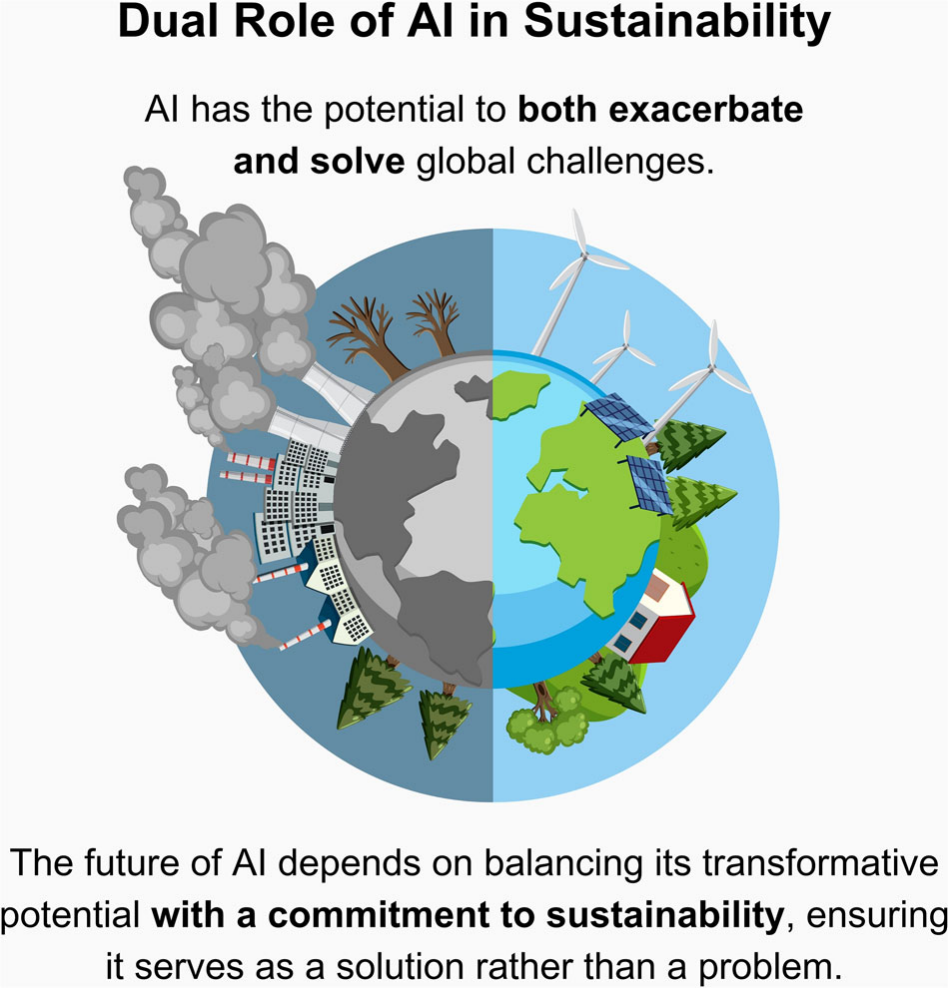
AI and sustainability paradox

Artificial intelligence (AI) is transforming the field of radiology, improving diagnostic accuracy, and enhancing workflow and operational efficiency [1]. A significant proportion of AI research [2] and the majority of AI-enabled medical devices approved by regulatory agencies (e.g., Food and Drug Administration or European Conformity) are related to radiology [3–5]. Many of these tools utilize deep learning (DL), a subset of AI techniques with higher computational requirements [6, 7]. Over time, these models have grown more complex, requiring larger sets of parameters to achieve improved performance. In the near future, foundation models, large-scale neural networks capable of processing multimodal data types (e.g., text, images, and audio) for versatile applications, will likely further expand the role of AI in radiology and medicine [8–10]. Yet this expansion reveals a paradox (Fig. 1). While AI drives remarkable innovation, it introduces considerable sustainability challenges, raising essential questions about how to balance technological advancement with broader responsibilities.

This situation parallels the steps of the Industrial Revolution, which increased global well-being and productivity, requiring the reconfiguration of entire industries, while also leaving a legacy of environmental damage and social inequalities [11]. Similarly, while AI promises exceptional advancements, it carries risks, including high energy consumption, increased carbon emissions, hardware waste, and unequal access to emerging technologies [12, 13]. Notably, energy used by information and communications technologies is projected to surpass 20–30% of global consumption by 2030 [14, 15]. Demand for AI services is also expected to grow by 30–40% annually over the next 5 to 10 years [16], further amplifying energy requirements. Addressing these issues requires integrating sustainability principles into the development and use of AI [17]. Rather than halting innovation, the goal would be to guide progress responsibly by adopting energy-efficient algorithms, embracing green computing strategies, and ensuring equitable and lasting technology distribution.

**Fig. 2:**
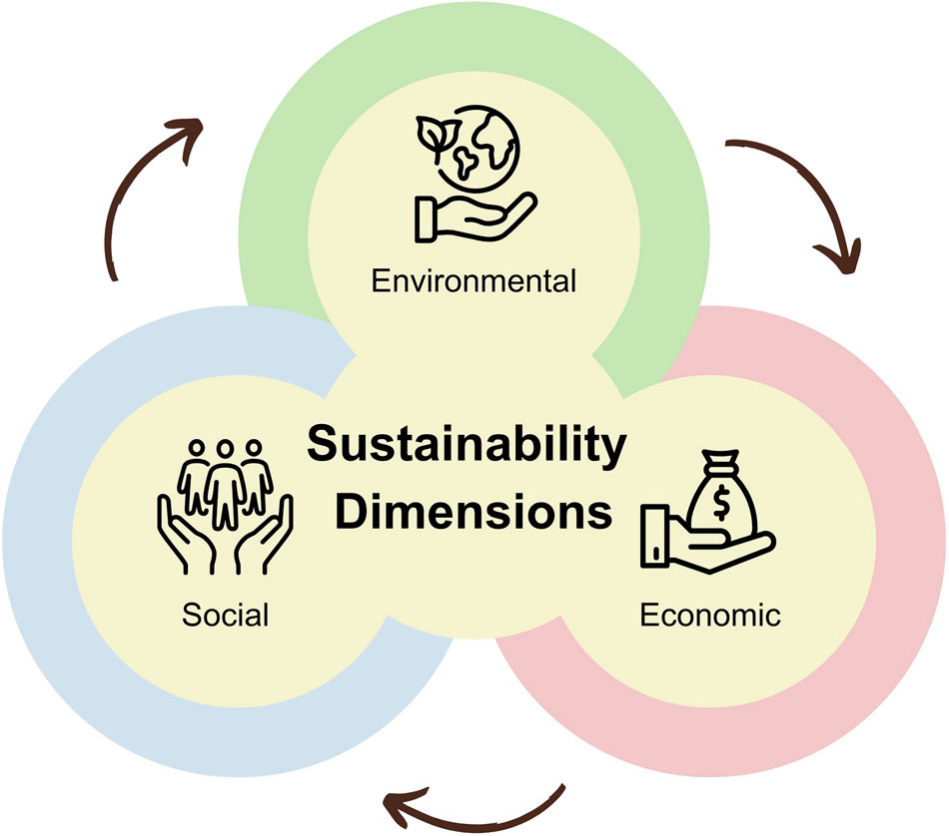
Three pillars of AI sustainability

Although sustainability is inherently multifaceted [17], previous research and reviews have primarily focused on environmental concerns within radiology AI [18, 19]. While not always immediately apparent, sustainability concerns in radiology extend beyond these issues. Therefore, this international collaborative review adopts a wider perspective, examining sustainability’s environmental, economic, and social dimensions, all of which ultimately also affect medical imaging (Fig. 2).

## Environmental sustainability

### Environmental impact and challenges

The process of training an AI model, particularly those based on DL, involves iterative computations across large datasets, often using high-performance hardware like graphics processing units (GPUs) or tensor processing units (TPUs). Each of these devices demands substantial energy, particularly when operating in parallel to reduce training times. The environmental impact extends beyond direct energy consumption by the hardware and its associated emissions to include indirect emissions from data center operations.

Once deployed, AI models continue to consume energy during inference, where algorithms analyze data to generate predictions, often exceeding that of training by an order of magnitude, with estimates ranging from hundreds to thousands of times higher. Wider adoption could further escalate inference-related energy demands. In radiology, this translates to real-time processing of imaging studies such as CT scans, MRIs, or X-rays, often requiring continuous operation of servers. The environmental cost becomes particularly concerning as the number of imaging studies per patient continues to rise in today’s healthcare, contributing substantially to global greenhouse gas emissions.

Taking the energy required for a single training run as an example, researchers analyzed the cumulative energy consumption of the top 20 AI systems in terms of carbon emissions published between 2021 and 2024 [20]. Total energy consumption per training run stands at 108 million kilowatt hours, indicating a carbon emission of 103 thousand metric tons of CO_2_-eq (carbon dioxide equivalent). To put this into perspective, the total CO_2_ (carbon dioxide) emissions from a single AI training run are equivalent to the daily carbon footprint of 7.5 to 8 million people worldwide (excluding aviation) [21, 22].

**Fig. 3:**
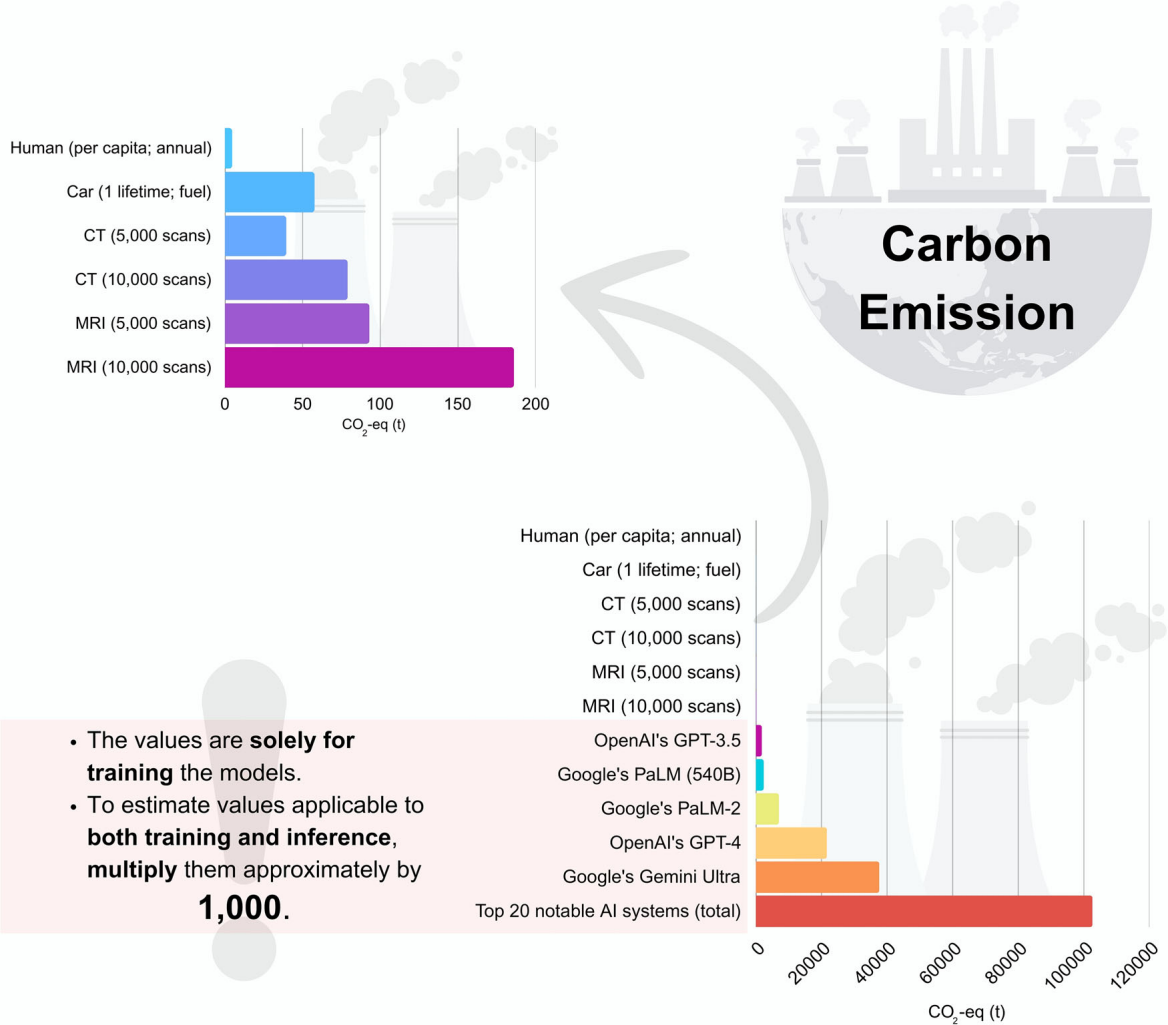
Carbon emissions of AI systems and relevant comparisons. Data compiled from multiple sources with necessary conversions applied [20–22, 98]. CO_2_-eq (t), carbon dioxide equivalent (metric tons)

Total carbon emissions from training and inference combined can be estimated to be 1000 times higher than a single training run, based on the inference-to-training energy consumption ratio [20, 23]. For context, training and deployment of a single large-scale AI model such as Google’s Gemini Ultra generates about 37.6 million metric tons of CO2-eq, comparable to around 7.5 million round trips from Tokyo to New York in economy class (direct flights, 1 person, Airbus 330) [20, 24, 25]. For reference, under the Paris Agreement, the lifetime budget per person on earth is around 50 tons CO_2_-eq [26]. When considering both training and inference, the energy usage of the top 20 AI systems results in around 103 million metric tons of CO_2_-eq emissions, which corresponds to the yearly carbon footprint of up to 22 million average people [21, 22]. This level of emissions is greater than many countries’ total annual emissions from energy in 2023, including Austria, Czech Republic, Romania, and Norway [27]. A visual comparison of carbon emissions is provided in Fig. 3.

Depending on country-specific regulations, the implementation of AI in radiology may rely heavily on cloud computing for storing and processing vast quantities of medical imaging data. This, in turn, increases the energy footprint of the systems managing these data. While cloud services are frequently regarded as more energy-efficient than on-premises solutions due to resource consolidation and optimized energy utilization across shared infrastructure, their overall sustainability is heavily dependent on the energy sources powering these facilities. This reliance has notable environmental implications, as data centers—integral to cloud infrastructure—are significant electricity consumers. Beyond powering servers, substantial energy is required to operate cooling systems that maintain optimal operating temperatures. It is estimated that data centers globally contribute approximately 1–2% of total electricity usage [28], with a potentially significant share associated with medical and scientific applications like AI-driven radiology.

Furthermore, water consumption for cooling these high-performance computing systems is a growing but often overlooked concern. Estimates suggest that global AI demand may account for 4.2 to 6.6 billion cubic meters of water withdrawal by 2027, 4–6 times the total annual water withdrawal of Denmark or half of the United Kingdom’s [29, 30].

The rapid advancement of AI technologies drives an ongoing need for more powerful and specialized hardware, also in hospitals and radiology departments, contributing to the growing global challenge of electronic waste (e-waste) [31]. The hardware components used for AI, such as GPUs, central processing units, and application-specific integrated circuits, are frequently retired before the end of their operational life due to the constant demand for faster, more efficient systems capable of handling AI’s computational intensity. These obsolete devices often end up as e-waste, which poses significant environmental hazards. Toxic substances such as lead, cadmium, and mercury in electronic components can contaminate soil and water, creating long-lasting ecological and health risks. Moreover, the manufacturing of AI hardware involves mining and refining rare earth elements like neodymium, tantalum, and cobalt, which are finite resources. These processes are energy-intensive and often associated with environmental degradation and significant carbon emissions.

**Fig. 4:**
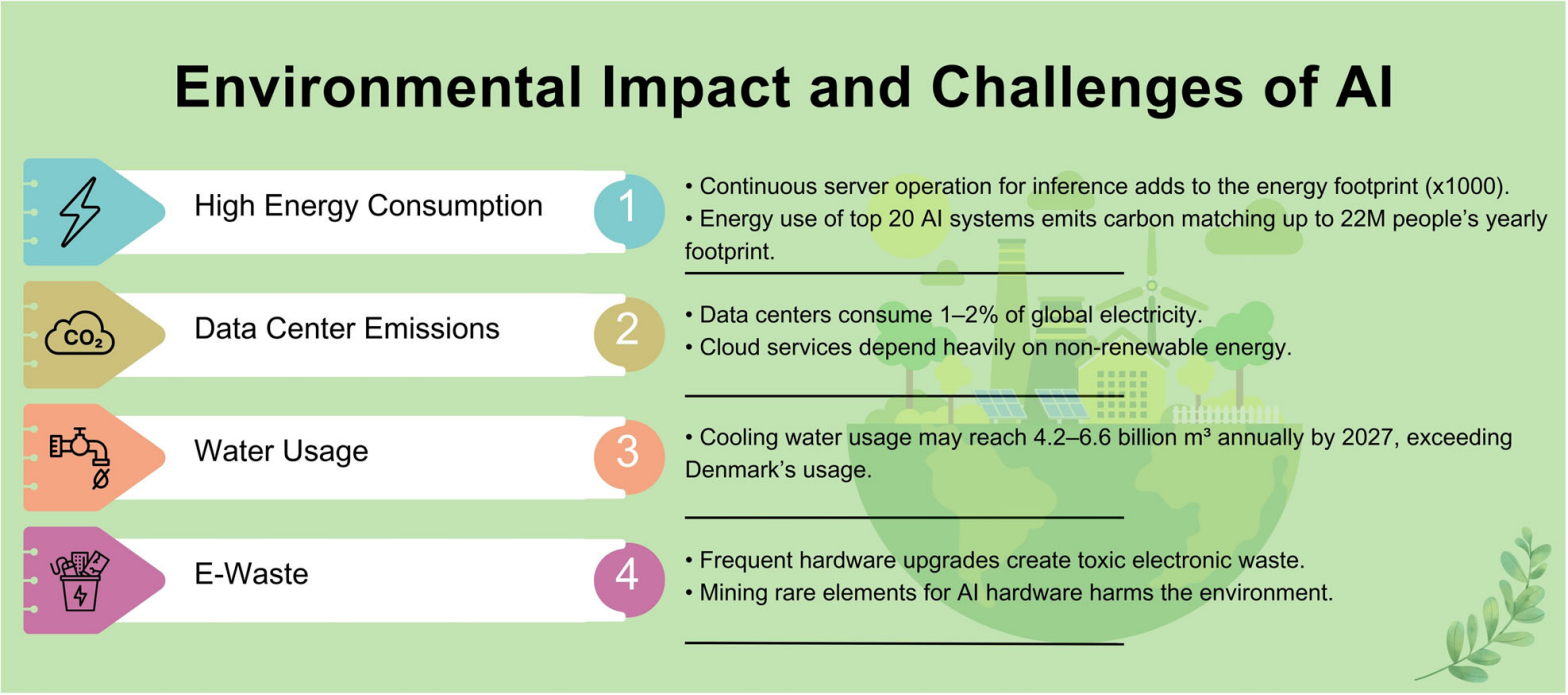
Environmental impact and challenges of AI

The environmental impact of AI, along with its associated challenges, is briefly outlined in Fig. 4.

### Strategies for environmentally sustainable AI

**Fig. 5:**
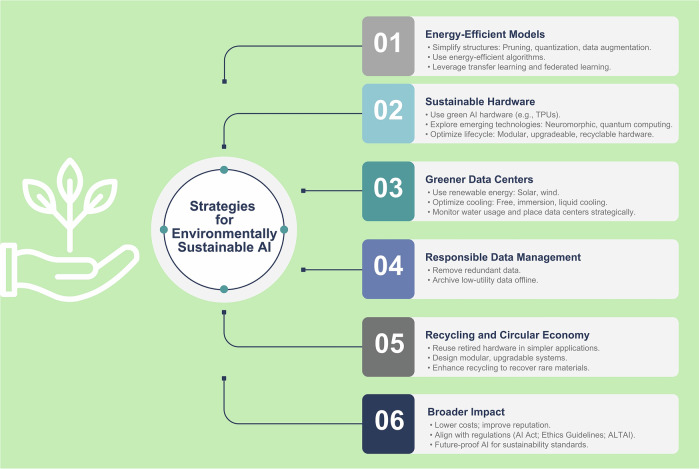
Strategies to mitigate environmental impact and challenges of AI. TPU, tensor processing unit; ALTAI, assessment list for trustworthy AI

Given the huge environmental impact of AI systems, there is a growing interest in developing and applying green AI practices that can act by various means (Fig. 5).

First, AI model energy consumption reduction is obtainable by simplifying the structure of the model itself. Several approaches are described for this purpose, including pruning, quantization, and data augmentation [32–35]. In addition, new algorithms can be trained to simulate the performance of larger models, with the difference of being also more energy-efficient. Distillation, creating a faster and energy-saver version of a “full” DL model, is an example of this procedure [36]. Probabilistic models can also be applied to approximate more complex computations, especially when obtaining high accuracy is not crucial [37]. Transfer learning, widely used in DL, is also helpful in reducing model training costs by fine-tuning pre-trained models [38]. In addition, federated learning may have huge benefits, enabling decentralized training without transferring large datasets. Beyond these, promoting a mindful approach that prioritizes meaningful innovation over the development of overly complex models aimed solely at publication can also be considered [39–41].

When first introduced, GPUs allowed the fast image processing of deep and machine learning models, thanks to their highly parallel architecture [42]. However, this high computational workload leads to high energy consumption and costs. Additionally, GPUs are limited by small memory capacity. To maximize the sustainability of AI models, green alternatives for AI hardware have been developed, such as TPUs and Field Programmable Gate Arrays [43, 44]. Neuromorphic and quantum computing are also emerging technologies reported as promising to overcome the limitation of DL in terms of lack of intralayer connections, need for manual configuration, and high energy consumption [45].

Data centers should consider alternative modalities to reduce energy consumption through exploiting renewable resources and optimizing cooling systems. Installation of solar panels and wind turbines has proved to reduce carbon emissions significantly [32, 46]. Innovative cooling technologies, such as free, immersion, and liquid cooling, are less energy-intensive compared to traditional methods [47, 48]. Integration of water footprint metrics that monitor and minimize water usage for cooling could further lessen the environmental impact of AI [29, 49]. The location of data centers is also strategic to maximize the efficiency of cooling systems, as centers located in colder geographic areas can significantly save energy costs [15].

Radiology departments can also play a role by implementing better data management practices. Reducing redundant storage, such as removing non-essential image reconstructions and archiving low-utility data offline, can contribute to more sustainable data storage practices in radiology [19]. Recycling programs and the circular economy for electronic components can further help mitigate this issue. For instance, reusing retired hardware in less demanding applications, designing modular hardware that can be upgraded rather than replaced, and improving recycling technologies to recover rare materials are practical steps toward reducing the lifecycle impact of AI hardware in radiology.

Beyond reducing climate impact, integrating green AI practices may bring direct benefits to data centers and their users, including medical imaging practitioners, primarily through lower energy costs. In this respect, guidelines were released in 2019 (Ethics Guidelines for Trustworthy AI) and 2020 (Assessment List for Trustworthy AI, ALTAI), highlighting the need for sustainable AI practices [50, 51]. More recently, the EU’s AI Act was approved in 2024 to “promote[s] the uptake of human-centric and trustworthy AI while ensuring a high level of protection of health, safety, fundamental rights” [52]. Particular attention is paid to applications considered at “high risk,” such as in the healthcare domain. Likewise, the U.S. released the “Executive Order on the Safe, Secure, and Trustworthy Development and Use of Artificial Intelligence,” aimed at including “principles, guidelines, priorities, and best practices aimed at ensuring the safe, responsible, beneficial, and sustainable global development and adoption of AI” [53]. Given these regulatory trends, implementation of sustainable AI practices will become a requirement while developing AI systems (Table 1).

**Table 1:** Hypothetical real-world scenarios illustrating selected strategies for environmentally sustainable radiology artificial intelligence (AI), along with hypothetical outcomes

Category	Scenario	Outcome
Energy-efficient training	A university hospital with a high-volume radiology department trains deep learning models for detecting abnormalities in chest X-rays. Given the high energy consumption of AI training, the information technology department adopts pruning and quantization techniques to optimize model efficiency. The hospital uses pruned models that remove redundant neurons and connections, reducing computational load. Quantization is applied to accelerate model inference while minimizing energy usage. Transfer learning is used to fine-tune pre-trained models instead of training new ones from scratch, significantly lowering carbon emissions.	A 40% reduction in energy consumption without significant loss in model accuracy.
Renewable energy integration	A diagnostic imaging center heavily relies on AI for automated tumor detection in MRI scans. To offset the high energy costs of AI inference, the center integrates solar panels on its rooftop. The center installs solar-powered battery storage to run AI servers during peak hours. AI model inference is shifted to nighttime when grid electricity demand is lower and renewable energy sources are more available. The center also partners with cloud providers using 100% renewable energy and carbon-neutral services.	The imaging center achieves a 25% reduction in carbon footprint and lowers operational costs.
Water-efficient data centers	A national radiology AI network uses cloud computing for AI-driven image analysis but faces concerns over excessive water use in cooling data centers. AI workloads are migrated to data centers in colder climates to reduce cooling-related water usage in which liquid and immersion cooling technologies are deployed instead of traditional air-based cooling. Water footprint monitoring tools are also used to optimize cooling water consumption and recycle used water where possible.	A 50% decrease in cooling-related water use, contributing to more sustainable AI operations.
Recycling and circular economy	A radiology department is upgrading its AI workstations, leading to potential e-waste from outdated GPUs and processors. Old hardware is repurposed for lower-intensity AI tasks, such as preliminary image preprocessing. The department partners with electronics recycling programs to properly dispose of AI chips and recover valuable materials like cobalt and rare earth elements. Modular hardware design is encouraged, where components such as RAM and storage can be upgraded instead of replacing entire systems.	75% of outdated hardware is repurposed or recycled, reducing electronic waste.
Optimization for sustainable clinical workflow	A tertiary hospital uses AI to prioritize critical cases in emergency radiology, such as stroke detection in CT scans. An energy-efficient triaging AI model is developed that only activates high-power computations when an abnormality is detected. AI tool is used to process some computations locally on imaging scanners instead of relying on cloud servers, reducing data transfer-related emissions. Batch AI processing is scheduled for non-urgent cases to optimize energy use during off-peak hours.	A 30% reduction in AI processing energy costs, faster turnaround times for urgent cases, and lower overall emissions.
Radiation dose optimization and energy efficiency	A radiology department implements an AI-powered radiation dose monitoring system to reduce unnecessary radiation exposure in CT and X-ray exams. AI analyzes historical scan data and automatically suggests lower radiation dose protocols without compromising image quality. Adaptive AI models optimize scan parameters based on patient-specific factors such as age and body mass to minimize radiation exposure. AI-powered quality assurance reduces scan repetition rates by detecting poor-quality images in real time.	A 20% reduction in patient radiation exposure and lower energy consumption for unnecessary repeat scans.

*GPU* graphics processing unit, *RAM* random-access memory

## Economic sustainability

### Economic impact and challenges

The integration of AI into radiology holds significant, but mostly retrospectively demonstrated benefits for diagnostic and operational capabilities. Currently, many value propositions marketed by AI providers emphasize efficiency improvements, such as reducing costs and workloads or enhancing clinical decision quality and patient care. However, these claims often rely on external legitimization strategies, such as obtaining legal approvals, forming partnerships with academic and medical institutions, and showcasing practical implementations, rather than providing robust evidence of systematic added value in clinical practice [54]. Marginal performance improvements, such as increasing sensitivity for abnormality detection by a few percentage points, have yet to meaningfully impact workload, operational efficiency, and patient-level outcomes [55].

The economic implications of AI adoption extend beyond license (or pay-per-use) and deployment costs [56]. AI systems must demonstrate long-term financial viability by, for example, reducing redundant imaging, improving patient outcomes, and supporting preventative care measures that lower overall healthcare costs. For instance, AI applications in early disease detection can potentially avert more costly interventions later, contributing to a more sustainable healthcare model. These long-term benefits have to be proven rather than assumed, either by prospective research or, if unattainable, by sophisticated health economic modeling (e.g., cost-effectiveness analysis) and, ideally, continuous monitoring.

Another key economic aspect is the rapid growth of AI as a booming industry. Although medical AI is a smaller niche within the broader market, its growth rate ranks among the highest [57]. This financial ecosystem often remains hidden from doctors, largely due to the lack of economic education in medical curricula, yet investments are the driving force behind innovation and the industry’s growth. However, this AI “gold rush” raises concerns that environmental, social, and governance considerations may be overlooked, as shareholder priorities currently seem to center on sales, even when products—beyond CE-mark certifications (from French, “conformité européenne,” meaning “European Conformity”)—lack sufficient clinical value to justify their price. Failing to address the economic sustainability of AI inadvertently increases healthcare costs, reducing accessibility for the general population, and potentially creating a new pharmaceutical-like industry driven by profit rather than equitable care. Such a scenario could exacerbate already existing inequities in healthcare delivery.

**Fig. 6:**
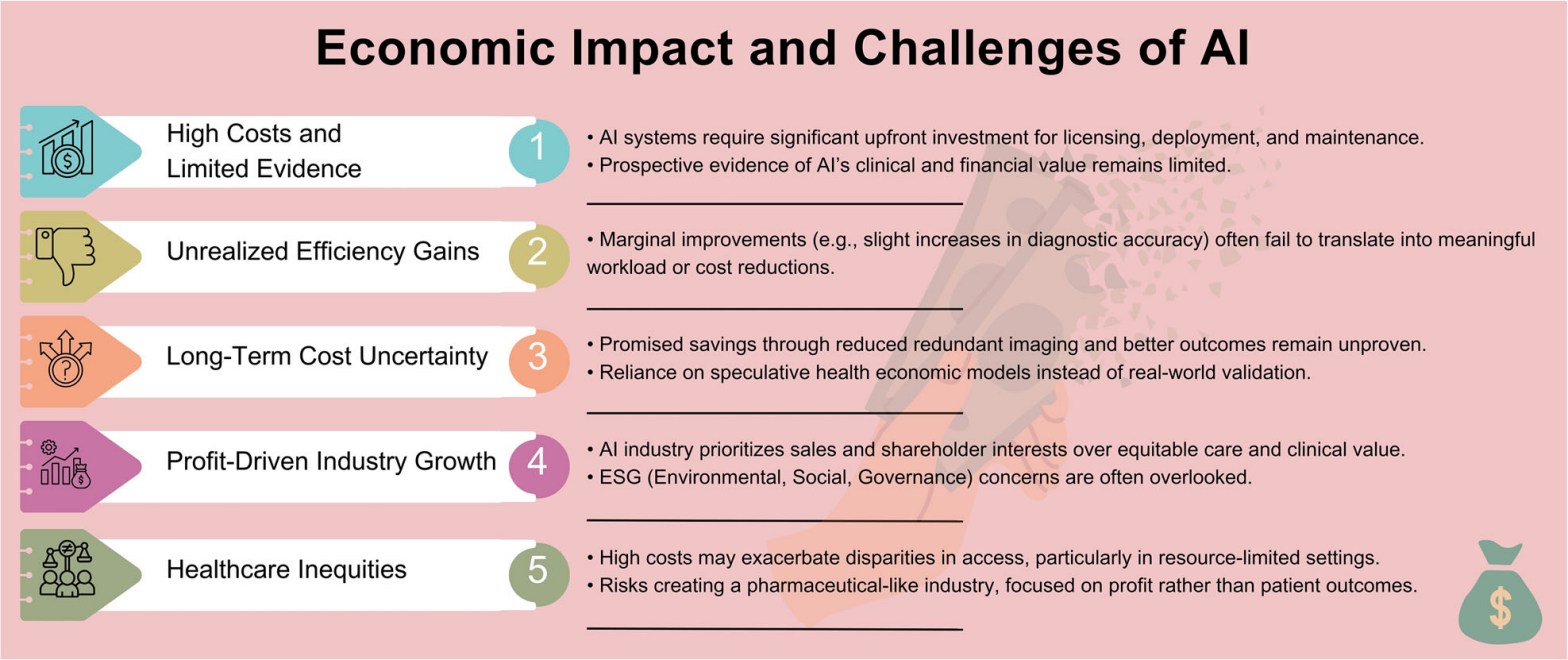
Economic impact and challenges of AI

Figure 6 summarizes the key economic impact of AI along with its associated challenges.

### Strategies for economically sustainable AI

For AI to justify its substantial license costs, which can roughly range from €10,000 to €100,000 annually for a single application, not even including the costs of deployment, its benefits must result in tangible improvements at the patient, institutional, and/or societal level. These could include measurable reductions in morbidity, such as lower interval cancer rates in mammography screening, improved functional outcomes in fracture detection tools, reduced reporting times, shorter emergency room waiting times, or reduced time to discharge. These quantifiable outcomes can then be translated to monetary value, either through metrics such as quality-adjusted life year (QALY) or by demonstrating a reduction in radiologist time spent per examination. For example, willingness-to-pay thresholds and incremental cost-effectiveness ratio (ICER) can then guide decisions on the cost-effectiveness of medical interventions. In the Netherlands, for example, these thresholds range from a maximum of €20,000–€80,000 per QALY gained, depending on disease severity. Radiologist time per exam can be assessed through median reading times, which is non-trivial and varies significantly across procedure types due to differences in complexity and workflow efficiency [58]. Such metrics can provide input for calculating return on investment and provide clarity on whether a particular application is economically viable for a healthcare center, independent of the broader question of who ultimately bears the cost [18].

**Fig. 7:**
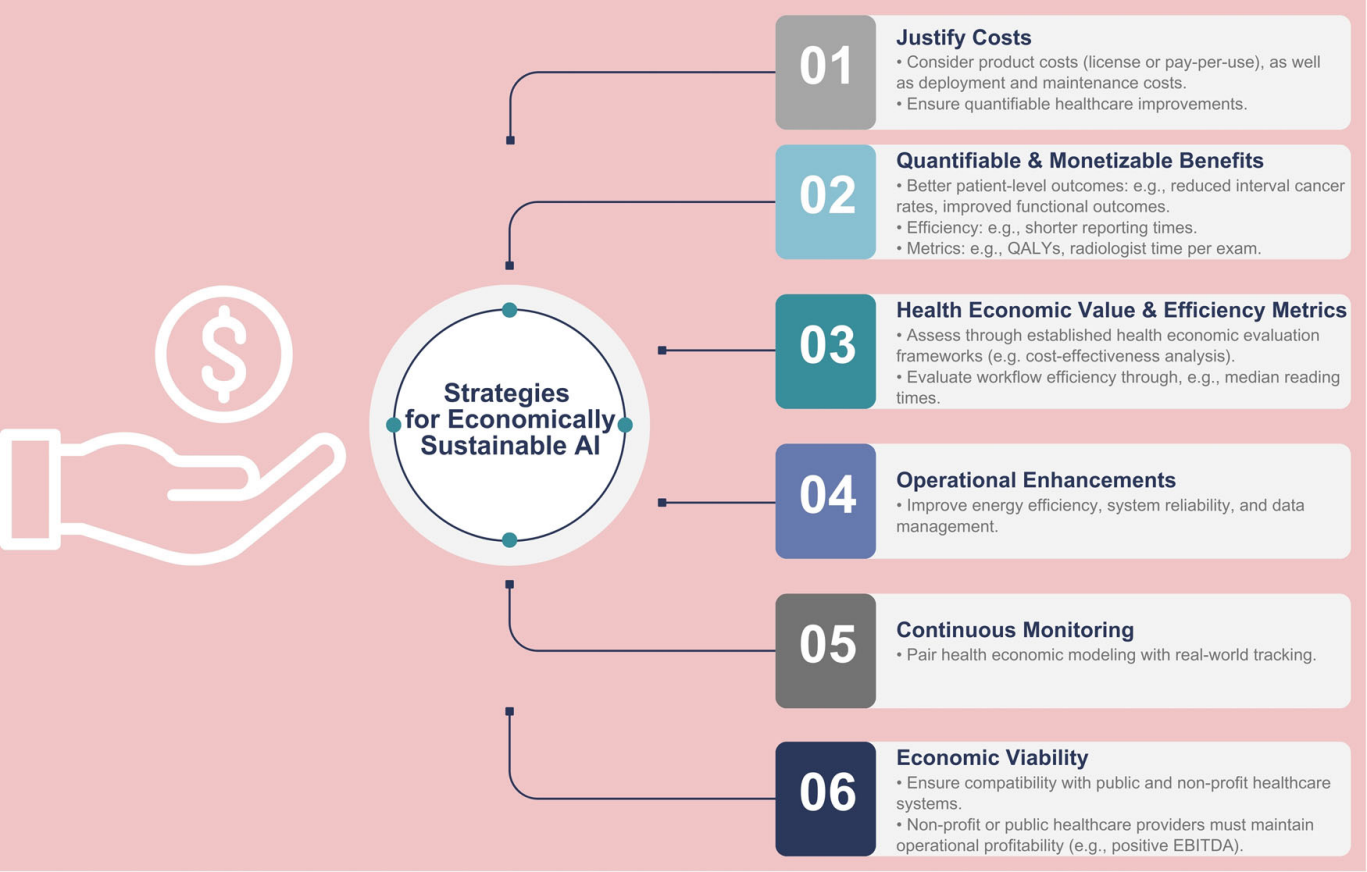
Mitigation strategies for the economic implications of AI. QALYs, quality-adjusted life years; EBITDA, earnings before interest, taxes, depreciation, and amortization

Although many healthcare centers in Europe are public or non-profit, those without heavy subsidies require a positive EBITDA (i.e., earnings before interest, taxes, depreciation, and amortization) margin to avoid stagnation, making economic sustainability crucial for the broader sustainability of healthcare systems. Beyond faster reporting times, operational enhancements such as improved energy efficiency, reduced downtime of imaging systems, and optimized data management through cloud integration are essential for ensuring the economic viability of AI-driven systems [18]. Moreover, prospective research and health economic modeling should be complemented by continuous monitoring to ensure the delivery of anticipated benefits. Overall, addressing these factors can strengthen the case for economically sustainable AI in radiology and healthcare (Fig. 7 and Table 2).

**Table 2:** Hypothetical real-world scenarios illustrating selected strategies for economically sustainable radiology artificial intelligence (AI), along with hypothetical outcomes

Category	Scenario	Outcome
Cost-effective implementation	A national healthcare system wants to introduce AI into public hospitals to solve reporting delays but faces budget constraints. To ensure economic viability, AI solutions are assessed for their impact on operational efficiency and patient outcomes. Health economic modeling was conducted before AI adoption. Priority was given to AI tools that demostrate cost-effectiveness compared to the standard of care.	10–15% savings in operational costs, justifying AI investment and ensuring sustainable implementation.
Reduction of redundant imaging	A large radiology department experiences a high rate of duplicate imaging requests, increasing costs and burdening radiologists. An AI-driven decision support system was deployed to alert physicians of redundant exams. AI was integrated to the electronic health records.	20% reduction in unnecessary imaging, leading to lower healthcare costs, reduced radiation exposure, and improved efficiency.
Operational efficiency	An emergency department struggles with long patient wait times due to radiology bottlenecks. An AI triage system is used to prioritize urgent cases, along with automated preliminary AI-based report generation. Turnaround times and potential cost savings were tracked to assess AI’s economic impact for 6 months.	30% reduction in emergency department turnaround times, leading to faster patient discharge and cost savings on hospital bed usage.
Value-based AI licensing models for hospitals	A mid-sized private hospital wants to adopt AI for radiology but struggles with high licensing costs. Hospital negotiates outcome-based AI contracts, opting for pay-per-use AI pricing models.	40% reduction in upfront AI investment costs and alignment of AI expenses with clinical benefits.

## Social sustainability

### Social impact and challenges

**Fig. 8:**
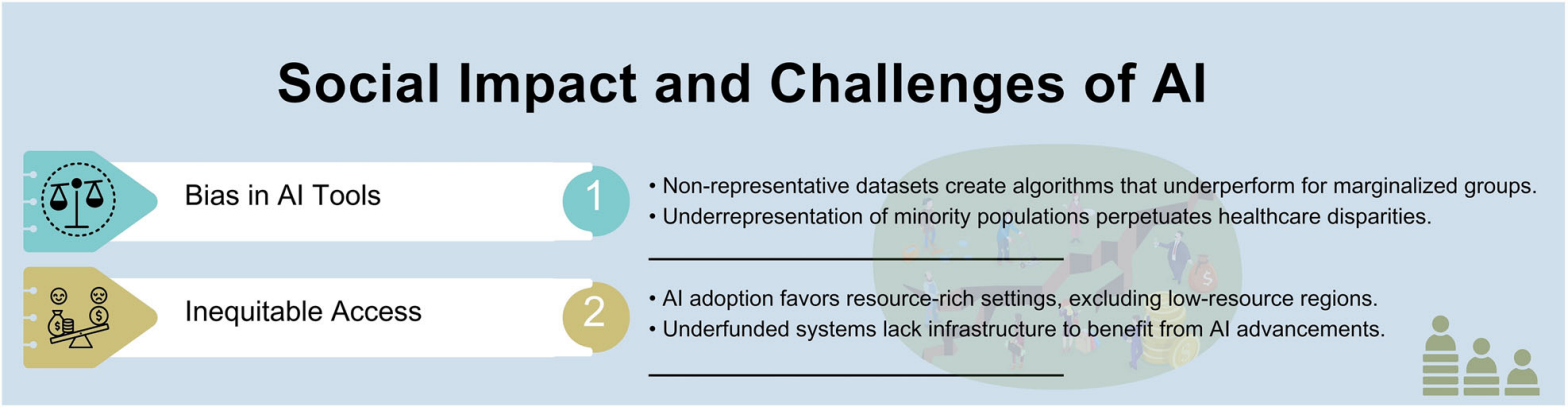
Social impact and challenges of AI

Social sustainability in technology refers to the development and use of tools that actively enhance the capacity of current and future generations to build healthy, equitable, and livable communities [59]. Social impact of AI with associated challenges is summarized in Fig. 8.

In AI specifically, the risk of bias that may disproportionately harm already marginalized groups is significant [13]. This bias often stems from non-representative datasets, incomplete data, or systemic inequities embedded in healthcare systems [13]. For example, the underrepresentation of minority populations in both the developers and training datasets can lead to algorithms that underperform for these groups, perpetuating existing disparities in healthcare delivery.

Ensuring equitable access to AI technologies is a core aspect of social sustainability. Currently, the adoption of AI solutions often favors resource-rich settings, leaving underfunded healthcare systems and low-resource regions with limited access to these advancements [60, 61]. This imbalance could exacerbate global health inequities, as regions without sufficient infrastructure are unable to benefit from AI-driven diagnostic and operational improvements.

### Strategies for socially sustainable AI

Policies aimed at democratizing access to AI tools through funding initiatives, partnerships, and scalable, cost-effective solutions are essential to prevent this divide. “Neocolonialism” must be avoided at all costs by ensuring that AI development and deployment involve inclusive collaboration with local stakeholders, prioritize culturally and contextually relevant solutions, and avoid imposing Western systems that fail to address the unique needs and priorities of underserved regions.

Transparent documentation of dataset composition and algorithmic processes is critical for achieving socially sustainable AI. The STANDING Together framework highlights the importance of accountability, urging developers to disclose dataset limitations and to proactively evaluate their algorithms’ impact on diverse populations [60, 61]. Continuous monitoring and robust and detailed post-deployment evaluations are needed to ensure that AI applications deliver equitable benefits across all populations and settings.

**Fig. 9:**
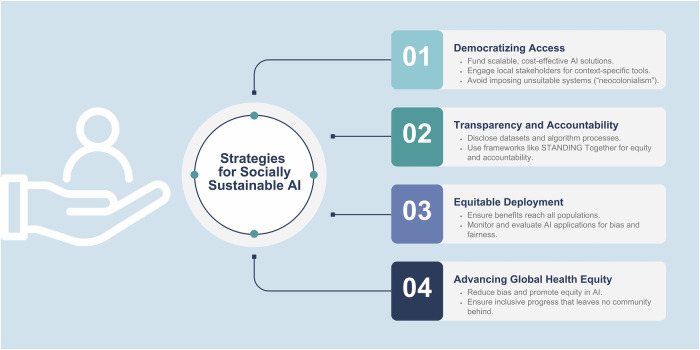
Strategies to mitigate social impact and challenges of AI

Strategies for socially sustainable AI are also summarized in Fig. 9 and examples relevant to radiology are given in Table 3.

**Table 3:** Hypothetical real-world scenarios illustrating selected strategies for socially sustainable radiology artificial intelligence (AI), along with hypothetical outcomes

Category	Scenario	Outcome
Equitable cancer screening in low-resource settings	A regional healthcare system in a low-income country struggles with limited radiologist availability, delaying breast cancer screening and diagnosis. AI-assisted mammography screening is deployed to help non-specialist healthcare workers interpret mammograms. Generalizability is ensured by training the AI on multi-ethnic (or local) populations. Partnerships with local governments, local community representatives, and non-governmental organizations are formed to assure adequate access for the local populations.	A 50% increase in screening coverage and earlier cancer detection in underserved communities.
Reducing racial and socioeconomic bias	A university hospital develops an AI model for lung nodule detection but finds that it underperforms in patients from minority populations. Bias audits are conducted by analyzing AI performance across different demographic groups. Training datasets are expanded to include more scans from historically underrepresented populations. The STANDING Together framework is also applied to ensure transparent reporting of AI fairness metrics.	A 25% improvement in AI accuracy for minority groups, ensuring more equitable diagnostic performance.
Addressing gender disparities	A global AI developer finds that its AI system for osteoporosis detection performs better in men than in women, raising concerns about gender bias. Training datasets are rebalanced to ensure equal representation of male and female patients. AI model parameters are adjusted to reduce bias in bone density measurements. Collaborations with medical researchers are established to validate model fairness across genders.	AI model achieves gender-balanced accuracy, ensuring fairer osteoporosis diagnosis for all patients.
Ethical AI development through community-driven partnerships	An AI company aims to launch a radiology AI tool in a very low-resource country but faces concerns about imposing Western-developed models without local adaptation. Local radiologists and healthcare professionals are involved in AI model development and further tuning. AI models are trained and tuned on region-specific imaging data to address unique disease presentations. Low-cost or subsidized AI licensing is offered to ensure affordability in low-resource settings.	Culturally and medically relevant AI solutions, improving AI adoption and healthcare outcomes without neocolonial overreach.
Explainability in AI for patient trust	Patients express skepticism about AI-assisted radiology diagnoses due to the “black box” nature of AI decision-making. Explainable AI tools are developed and incorporated that show visual heatmaps highlighting regions of interest in medical images. Clear patient-friendly reports are provided, explaining AI-generated findings alongside radiologist interpretations. Community outreach programs are conducted to educate the public about AI’s role in healthcare.	Higher patient trust in AI diagnostics, as demonstrated by measurable improved acceptance and adherence to medical recommendations.
Accountability through continuous monitoring	A hospital network adopts AI for stroke detection in CT scans but wants to ensure long-term fairness and effectiveness. Ongoing AI performance audits are established to track disparities in model performance across different patient populations. Real-world monitoring tools are implemented to flag potential biases as AI systems are used in practice. AI vendors are required to provide transparent documentation of model updates and retraining processes.	AI systems remain fair, reliable, and effective across diverse patient demographics, reinforcing social sustainability.

## AI for more sustainable radiology

**Fig. 10:**
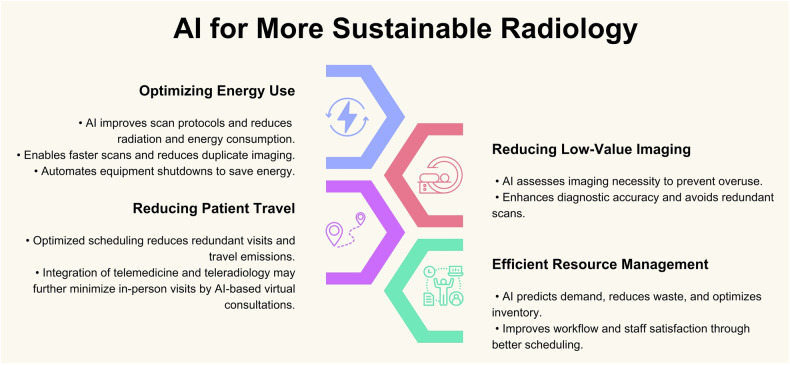
Key opportunities for utilizing AI to achieve sustainability in radiology

Key opportunities for utilizing AI to achieve sustainability in radiology are summarized in Fig. 10.

### Optimizing scan protocols and reducing energy consumption

Traditionally, radiologists have determined scan protocols based on factors such as clinical indication and patient characteristics [62]. While effective, this manual approach has inherent limitations in scalability, consistency, and energy consumption. AI-based tools can address these challenges by enabling intelligent protocoling for most radiologic examinations and improving quality and standardization [63]. In CT, AI systems can dynamically adjust imaging parameters, such as tube current and voltage, to tailor scans to each patient, reducing radiation exposure while also minimizing energy use [64]. Furthermore, technologies such as AI-assisted compressed sensing have revolutionized MRI efficiency by significantly reducing scan times while maintaining high image quality, thus directly contributing to lower energy consumption per scan [65, 66]. Notably, such AI tools and beyond can also be effectively applied to low-field-strength MRI systems, which are both more accessible and environmentally friendly due to reduced greenhouse gas emissions during both the manufacturing and operational phases [67].

AI can also improve energy efficiency in radiology by automatically shutting down equipment during idle periods and starting up systems at optimal times [68]. Additionally, it can be used to monitor other energy-intensive devices in radiology departments, such as PACS workstations, and automatically power them down during periods of inactivity to reduce unnecessary energy consumption [69].

### Reducing redundant and low-value imaging, radiation exposure, and contrast usage

A more recent application of AI in radiology involves generating synthetic CT images from MRI scans. This approach could eliminate the need for an additional CT scan, streamlining the radiation treatment planning process in cancer patients [70].

AI has the potential to significantly reduce low clinical-value imaging by providing tools to improve decision-making, prioritize necessary imaging studies, and filter out unnecessary ones. Patient-specific data, including medical history, lab results, and body size, can be analyzed, providing real-time decision support that helps assess whether imaging is necessary [71–73]. By identifying requests that may not offer significant clinical benefit, AI helps optimize imaging resources and reduce overuse.

Although intravenous contrast agents are typically well-tolerated, it is important to minimize their use to enhance patient safety and reduce costs and waste [74]. AI-powered imaging sequences have demonstrated the ability to lower the required contrast dosage without compromising diagnostic accuracy, offering both clinical and environmental benefits [75].

### AI-driven workflow optimization and scheduling

Conventional scheduling methods in radiology often result in inefficiencies such as overlapping appointments, underutilized equipment, and redundant visits, which can increase patient travel and reduce resource utilization efficiency [76]. AI-driven scheduling systems present an opportunity to address these challenges by optimizing appointment allocation to align with patient locations, imaging modality availability, and staffing schedules, thus decreasing unnecessary travel and wait times [77, 78]. This is particularly relevant in modalities such as CT or MRI, where the duration of imaging studies is influenced by specific protocols. ML algorithms may enhance this process by predicting factors such as expected wait times, and patient no-shows based on patient-specific and environmental variables [79]. Additionally, AI tools can facilitate contingency planning by dynamically managing disruptions, such as scanner downtime, prolonged examinations, or late arrivals, ensuring smoother workflows and better utilization of imaging resources [80].

### Telemedicine and teleradiology for reduced travel and emissions

The integration of telemedicine and teleradiology further enhances these advancements by offering patients the option of virtual consultations, expert second opinions, or simple explanations of imaging results. This approach significantly reduces the need for in-person visits, thereby lowering travel-related emissions and minimizing patient inconvenience [81]. AI chatbots might also be useful in addressing patients’ requests [82, 83].

### Improving image quality and reducing repetitive scanning

High-quality medical imaging is essential for accurate diagnosis, effective treatment planning, and optimal patient outcomes [84–86]. It plays a critical role in preventing misdiagnosis, minimizing redundant imaging, and ensuring timely intervention, particularly in critical care scenarios [87, 88]. AI algorithms can automatically detect and correct issues such as motion artifacts, noise, or blurring, enhancing image clarity and diagnostic utility in modalities like CT and MRI, which is especially beneficial in challenging cases such as low-contrast tissues or small lesions [89, 90].

### AI-driven resource management and demand forecasting

Resource management and demand forecasting are often hindered by the limitations of traditional methods, which struggle to adapt to rapidly changing needs. In radiology departments, this can result in inefficiencies such as overstocking or shortages of contrast media, emerging drugs, and all imaging-related disposable materials [91, 92]. Integrating AI offers a transformative approach by enabling precise demand prediction, streamlined workflows, and effective inventory management, thereby reducing waste and enhancing resource utilization [93, 94]. An ongoing challenge within the medical departments is the shortage of staff, excessive workloads, and high turnover rates, which contribute to employee dissatisfaction [95]. Self-rostering, a scheduling system that allows employees to create their own work schedules, offers a potential solution by shifting the responsibility of work schedule creation to employees, allowing them greater control over their working hours. Integrating AI tools into this process can streamline roster generation, minimize the time spent on adjustments, and ultimately reduce the costs associated with scheduling [96].

### Optimizing equipment utilization and maintenance planning

Through analysis of historical data, AI can uncover patterns that contribute to the overuse or underuse of specific modalities, aiding radiology departments in optimizing decisions related to equipment procurement and maintenance planning. Additionally, the adoption of cloud-based technologies and remote collaboration platforms facilitates workload sharing among radiologists, streamlines resource management across various sites, and alleviates operational bottlenecks. These advancements not only enhance workflow efficiency but also minimize resource wastage and improve overall cost-effectiveness [97].

## Call for action and future directions

AI plays a dual role in sustainability. It has the potential to drive innovation, streamline workflows, and reduce resource consumption, thereby contributing to more efficient and sustainable practices. However, its significant energy demands and broader environmental, economic, and social impacts cannot be overlooked. Striking a balance between leveraging AI’s transformative capabilities and adopting sustainability practices is crucial for shaping a responsible future in radiology.

**Fig. 11:**
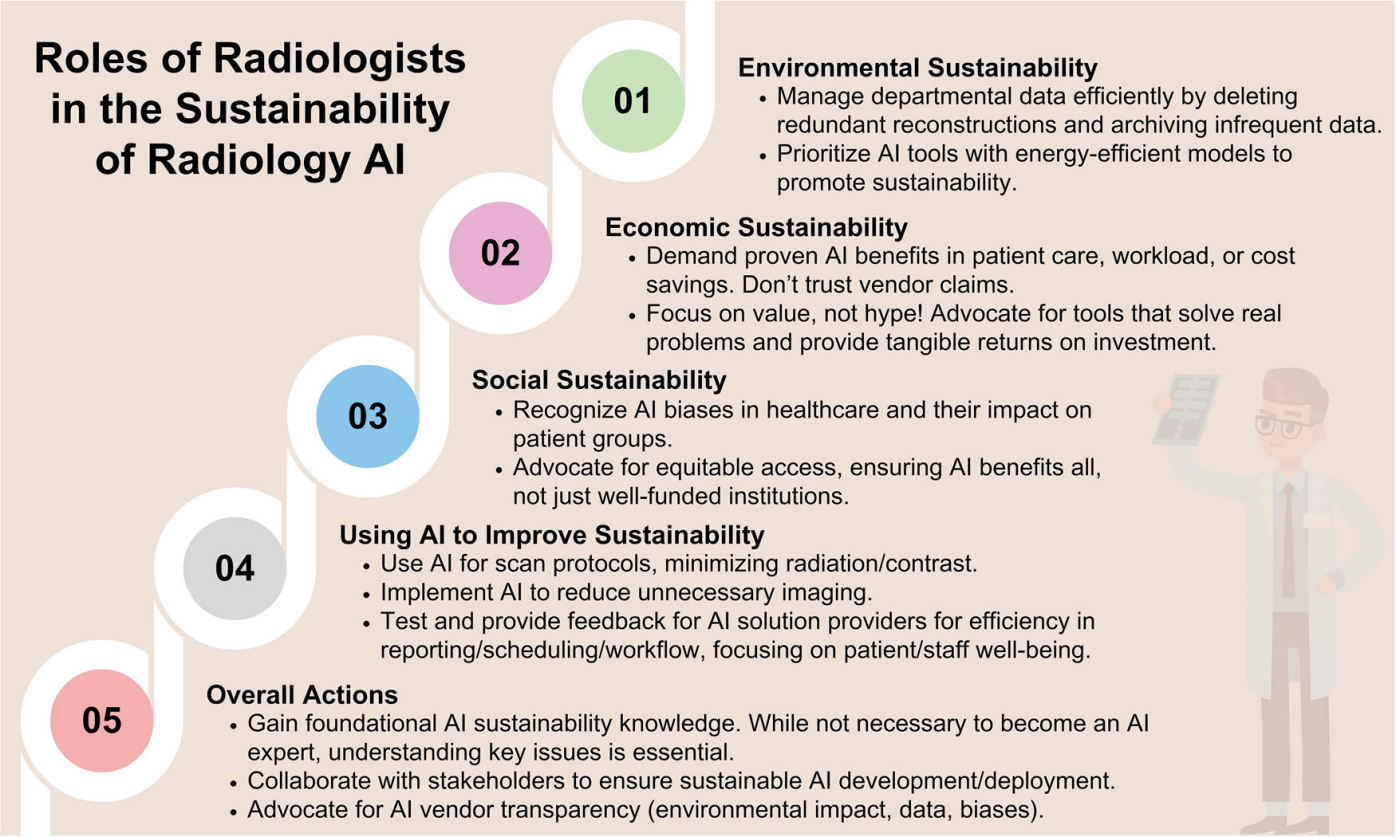
Potential roles of radiologists in sustainability of radiology AI

A fundamental shift towards sustainability is imperative within the radiology AI community. A summary of the potential roles of radiologists in the sustainability of radiology AI is presented in Fig. 11. Importantly, radiologists should acquire fundamental knowledge of sustainability. While becoming an AI expert is not necessary, understanding key sustainability issues is essential in this regard. It is also crucial to acknowledge that many challenges and solutions lie beyond the responsibility of radiologists, requiring engagement from various and higher-level bodies. However, addressing these challenges demands a collective commitment from radiologists, AI scientists, industry partners, and policymakers to integrate environmental, economic, and social considerations into the development and deployment of AI technologies. In this regard, the adoption of “green AI” principles [15], advocacy for supportive policies, and conscious decision-making in research and procurement are crucial to ensure equity and access while mitigating environmental impacts.

**Fig. 12:**
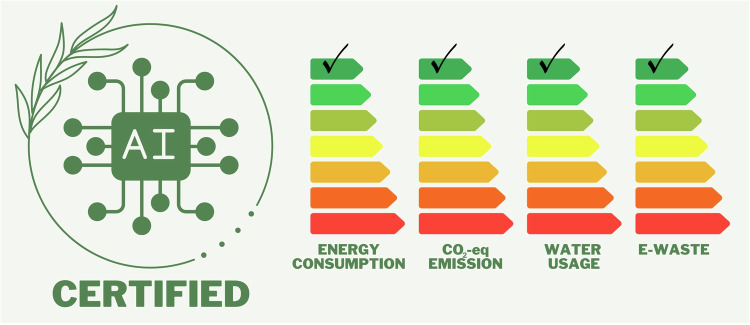
Ecolabel mock draft for AI tools. CO_2_-eq, carbon dioxide equivalent

Current methods for evaluating the sustainability of AI systems in radiology are inadequate, primarily focusing on carbon emissions and lacking consistency [15, 18, 19]. Standardized methodologies and tools are needed to accurately measure and report the impact of AI [17]. These methods should encompass all three dimensions of sustainability, address potential biases, evaluate accessibility for diverse populations, and consider the economic implications of AI deployment. The development of a user-friendly radiology AI ecolabel (Fig. 12), analogous to Energy Star, could facilitate stakeholder assessment and incentivize sustainable practices [18].

Research should prioritize the development of “green-in AI” algorithms that are inherently energy-efficient [15]. Strategies to reduce computational cost, such as model compression and specialized hardware, should be explored. Furthermore, ensuring social equity and economic viability is essential. This involves addressing algorithmic biases, promoting equitable access to AI-enhanced care, and considering the economic impact on healthcare systems and the workforce. The potential of “green-by AI” solutions, where AI addresses sustainability challenges within radiology, also warrants investigation.

Comprehensive policy frameworks are necessary to guide the sustainable development and deployment of radiology AI. These frameworks should address all dimensions of sustainability, promote transparency and accountability, and incentivize responsible innovation [17]. The European Union’s AI Act provides a model, but global initiatives are needed. Addressing these challenges requires transparency, and interdisciplinary collaboration among radiologists, AI scientists, ethicists, policymakers, and industry stakeholders. Educational initiatives are essential to raise awareness within these communities, including radiology. Future research should also investigate the interdependencies between the sustainability dimensions of AI systems and adopt a holistic approach to mitigate potential systemic risks associated with AI deployment [17].

## Conclusion

Radiology is at the forefront of AI innovation in healthcare, yet the paradoxical nature of AI highlights the importance of informed decision-making at individual, institutional, and collective levels. Ensuring these transformative technologies align with principles of sustainability is paramount. By mitigating AI’s environmental, economic, and social impact, we can harness its tremendous transformative potential while safeguarding planetary health. This necessitates a collective commitment to sustainable AI practices, ensuring that radiology AI truly serves society, protects the environment, and provides long-lasting benefits for future generations.

## Abbreviations

AI: Artificial intelligence
CO_2_: Carbon dioxide
CO_2_-eq: Carbon dioxide equivalent
DL: Deep learning
GPU: Graphics processing unit
QALY: Quality-adjusted life year
TPU: Tensor processing units
